# Parenting Programme for Child Development

**Published:** 2007-03

**Authors:** Patrice L. Engle

**Affiliations:** Department of Psychology, Cal Poly State University, San Luis Obispo, CA 93407, USA, Email: pengle@calpoly.edu

Over 200 million young children in the developing world do not achieve their potential for cognitive and social-emotional development due to the combined risks of poor health, poor nutrition, and absence of supportive learning and nurturing environments (1). Aboud's study is an important contribution to learning how to reduce these risks (2), and there is an urgency for this knowledge. With globalization, urbanization, and universal schooling, the loss of potential is resulting in ever-increasing disparities and inequalities. Poorer and disadvantaged children enter school less prepared, progress more slowly through school, and may learn less. Rarely can these children make up the differences in learning achievement that existed when they entered school. Decades ago countries, such as the US, India, and Peru, recognized that young children from poorer backgrounds need a head start before school.

The evidence that early interventions can stem this loss in developmental potential is now strong (3, 4). Heckman argues that the only period of a child's life in which an intervention is likely to be cost-effective is during early childhood (5). Herrod shows that early interventions affect a person's health and development throughout the life course and suggests that the health system should play a role not only in child survival, but also child development (6). The evidence is clear; but the action is often lacking.

Why has the implementation of early child-development programmes been slow? Problems include overlapping responsibilities of different ministries and the lack of a single global measure of child development to track progress, but most important is the lack of a single agreed-upon ‘package’ of interventions. The best intervention may depend on the particular situation.

A recent review of evaluated programmes in developing countries found that effective interventions at scale exist (3). The impact was more consistently significant for direct services to children, e.g. quality childcare programmes, than for parenting programmes which impact children indirectly. Yet, these parenting programmes have a greater outreach, can reach children in the critical first three years of life, and are more likely to be consistent with traditional childrearing. Logically improving parenting skills would seem to be the most cost-effective and sustainable strategy for supporting young children's development. However, we do not yet have enough research on parenting programmes in developing countries to identify what makes them effective—or not.

Aboud's paper is a welcome and much-needed assessment of what works and what does not work in parenting programmes, with practical and theory-based suggestions for improvement (2). The paper carefully shows that a high-quality intervention resulted in significant changes in parent knowledge and in play materials provided by the mother, but not in child nutritional status or cognitive development. This paper makes a critical point: a parenting intervention, however well-intentioned and well-prepared, that does not give families the opportunities to practise and try out new skills is unlikely to be effective in changing children's behaviour. Theories of behaviour change have come to the same conclusion.

How can an intervention change parents' knowledge but have no impact on children? Parents learned the information but apparently did not apply the knowledge. They provided some play materials but had not developed ways to interact in a responsive and stimulating way with their children. Specific practice opportunities, especially with their own children, would probably have made a difference. The intensity of the intervention may also have been too low, since parents attended on average only 16 of the planned 40 sessions. In the future, programmes should provide families opportunities to test out and practise new ideas for play and responsive communication with their children, and the discussion should build on their belief systems about child development.

Progress has been made because an NGO—Plan International—was willing to evaluate and improve a parenting programme, and a research organization—ICDDR,B—was willing to support the efforts. All should be congratulated for this effort. Rarely in the rush of setting up programmes is there time and funding for programme evaluation. However, this is a critical contribution to better programming, and all international agencies including NGOs should be encouraged—even required—to include an evaluation component that can improve quality. While it is often hard to get funds for doing an evaluation, putting this component—as much as 15%—in the beginning is extremely important. Informed policy requires evidence.

The most effective parenting programmes are not limited to one sector. Many organizations develop parenting programmes, only some of which have a holistic approach to young children. UNICEF, for example, assists as many as 60 countries in parenting and focuses on child survival, child growth and development. This holistic approach is consistent with a family's goal that their child should not only survive but also develop well. However, despite successes, too often parenting programmes show only modest effects for children. We must continue to learn about the best strategies for helping parents care for their young children, particularly during the first three years when the basis for learning and social development is being formed. Developing effective interventions for supporting families to better prepare children should be the highest priority. Only with developing-country research on what makes a parenting programme effective will we be able to give all children the best start in life. There can be no delay.

## References

[1] Grantham-McGregor S, Cheung YB, Cueto S, Glewwe P, Richter L, Strupp B, International Child Development Steering Group (2007). Developmental potential in the first 5 years for children in developing countries. Lancet.

[2] Aboud FE (2007). Evaluation of an early childhood parenting programme in rural Bangladesh. J Health Popul Nutr.

[3] Engle PL, Black MM, Behrman JR, Cabral de Mello M, Gertler PJ, Kapiriri L (2007). Strategies to avoid the loss of developmental potential in more than 200 million children in the developing world. Lancet.

[4] Walker SP, Wachs TD, Gardner JM, Lozoff B, Wasserman GA, Pollitt E (2007). Child development: risk factors for adverse outcomes in developing countries. Lancet.

[5] Heckman JJ (2006). Skill formation and the economics of investing in disadvantaged children. Science.

[6] Herrod HG (2007). Do first years really last a lifetime?. Clin Pediatr.

